# The impact of physiological metabolite levels on serine uptake, synthesis and utilization in cancer cells

**DOI:** 10.1038/s41467-021-26395-5

**Published:** 2021-10-26

**Authors:** Marc Hennequart, Christiaan F. Labuschagne, Mylène Tajan, Steven E. Pilley, Eric C. Cheung, Nathalie M. Legrave, Paul C. Driscoll, Karen H. Vousden

**Affiliations:** The Francis Crick Institute, 1 Midland Road, London, NW1 1AT UK

**Keywords:** Cancer metabolism, Cell growth

## Abstract

Serine is a non-essential amino acid that is critical for tumour proliferation and depletion of circulating serine results in reduced tumour growth and increased survival in various cancer models. While many cancer cells cultured in a standard tissue culture medium depend on exogenous serine for optimal growth, here we report that these cells are less sensitive to serine/glycine depletion in medium containing physiological levels of metabolites. The lower requirement for exogenous serine under these culture conditions reflects both increased *de novo* serine synthesis and the use of hypoxanthine (not present in the standard medium) to support purine synthesis. Limiting serine availability leads to increased uptake of extracellular hypoxanthine, sparing available serine for other pathways such as glutathione synthesis. Taken together these results improve our understanding of serine metabolism in physiologically relevant nutrient conditions and allow us to predict interventions that may enhance the therapeutic response to dietary serine/glycine limitation.

## Abstract (web summary)

Cancer cells in culture are often grown in media conditions containing unphysiological metabolite levels. Here, the authors grow cells under physiological metabolite levels to further understand the reliance of cells on serine and find that when grown under these conditions the cells are less sensitive to serine withdrawal.

## Introduction

Numerous studies have explored the potential of dietary interventions for cancer therapy and an increasing understanding of the metabolism of cancer cells has led to the development of therapeutic strategies focused on the dietary depletion of selected nutrients^1,2^. Serine, a non-essential amino-acid implicated in numerous different metabolic processes such as antioxidant defence, one-carbon metabolism and de novo nucleotide synthesis, has been identified as a key nutrient to support tumour cell growth in vitro and in vivo^3,4^. Mice fed with a serine/glycine-depleted diet show significant reduction of circulating levels of serine, leading to a reduction of tumour growth and increased survival in xenograft and genetically engineered mouse models of cancer^5–9^. However, the sensitivity of tumours to dietary serine depletion can be modulated by additional genetic alterations. Cancers arising following amplification of phosphoglycerate dehydrogenase (PHGDH), the first enzyme in the de novo synthesis pathway for serine, for example, can be more resistant to lack of serine in the diet while loss of one of the serine synthesis pathway (SSP) enzymes can increase sensitivity to dietary serine depletion^8^. The SSP can also be activated by several common oncogenic alterations such as activation in KRAS while loss of p53 can make tumours more sensitive to a reduction in circulating serine^3,6,7,10^.

In light of the success of dietary intervention approaches in mouse models, there has been an increased interest in translating this research into patients in order to improve the response to cancer therapies^1,2^. However, most of the in vitro studies examining responses to nutrient deprivation have been carried out using classical cell culture media, which were formulated half a century ago with the aim of deriving and easily maintaining mouse or human cell lines in culture^11^. These formulations did not attempt to recapitulate the human metabolic environment and lack some metabolites present in human circulation while containing an over-abundance of others^12^. Recently, there has been increasing evidence that nutrient levels in culture media can influence the metabolic phenotype and even the response to targeted therapies^13,14^. This has led to the development of culture techniques and tailored media that better represent in vivo environments such as the brain or the blood circulation^15–18^. These studies have resulted in the formulation of two physiologically relevant media with similar compositions, Plasmax and HPLM^16,17^. In breast cancer cells, the high level of pyruvate available in DMEM compared to Plasmax, has been shown to stabilise the hypoxia inducible factor 1a (HIF1a) leading to an hypoxic response even under normoxia^16^. In addition, the high level of arginine in DMEM was shown to reverse a reaction in the urea cycle catalysed by the arginosuccinate lyase^16^. This response was not observed when cells were grown in Plasmax, which contains lower, physiological levels of arginine. Untargeted metabolomics also revealed that spheroids grown in Plasmax were metabolically more similar to breast tumours than spheroids grown in classical media^16^. Additionally, urate, the end product of nucleotide catabolism, is a metabolite that is present in human circulation but absent in conventional cell culture media. Interestingly, urate present in HPLM can inhibit UMP synthetase (UMPS) and so regulate pyrimidine synthesis. UMPS is also responsible for the metabolism and cytotoxicity of 5-FU, and cell cultured in HPLM were shown to be less sensitive to 5-FU due to the presence of urate^17^. Another recent study using CRISPR screens to identify genetic dependencies in blood cancer cells revealed a number of genes that were selectively essential in HPLM^19^. While the physiological media do not fully recapitulate the complexity of a human tumour microenvironment, these studies show that useful information about the response of cells to metabolic interventions can be obtained using these media conditions.

There has been considerable interest in the potential for serine limitation during cancer therapy, and we have used Plasmax to more fully understand how cancer cells respond to serine limitation in patients and to identify potential targets that could enhance the efficacy of dietary serine starvation. In this work, we observe that cells grown in Plasmax are less sensitive to serine/glycine deprivation due to a multi-layer rewiring of serine metabolism in these more physiologically relevant conditions.

## Results

### Serine and glycine metabolism in Plasmax

**Fig. 1:**
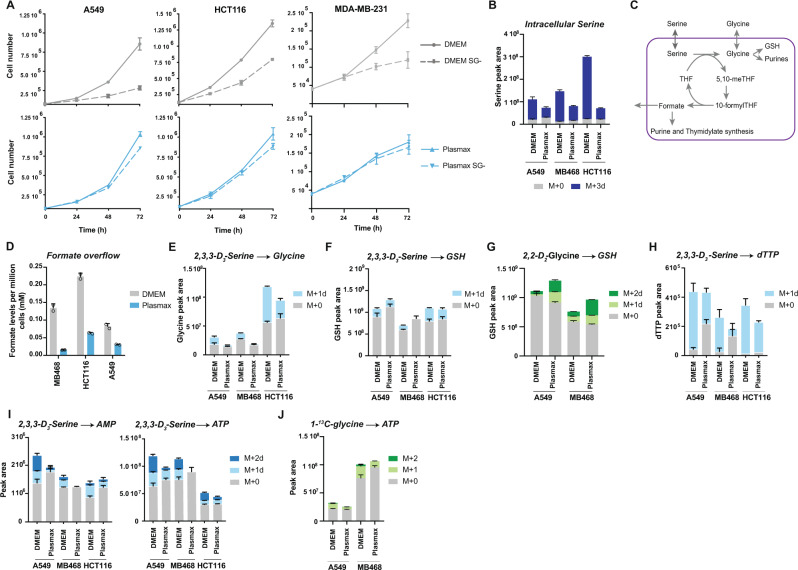
Serine and glycine metabolism in Plasmax. **A** Growth curve of A459, HCT116 and MDA-MB-231 grown in DMEM and Plasmax with or without serine/glycine (SG-). Data are represented as mean ± SD of triplicate wells. **B** Intracellular levels of serine measured by LC–MS after incubation with 400 µM or 140 µM of 2,3,3-D_3_-Serine in DMEM and Plasmax, respectively. Data are represented as mean ± SD of triplicate wells. **C** Serine is converted by the SHMT enzymes into glycine which will contribute to a number of important processes such as redox defence, nucleotide production and provision of one-carbon units to the folate cycle and methylation reactions. **D** Extracellular formate measured after 72 h in DMEM (grey) and Plasmax (blue) by NMR. Data are represented as mean ± SD of duplicate wells. **E** Intracellular levels of glycine measured by LC–MS after 4 h incubation with 400 µM or 140 µM of 2,3,3-D_3_-Serine in DMEM or Plasmax, respectively. Data are represented as mean ± SD of triplicate wells. **F** Intracellular levels of GSH measured by LC–MS after 4 h incubation with 400 µM or 140 µM of 2,3,3-D_3_-Serine in DMEM or Plasmax, respectively. Data are represented as mean ± SD of triplicate wells. **G** Intracellular levels of GSH measured by LC–MS after 4 h incubation with 400 µM or 330 µM of 2,2-D_2_-Glycine in DMEM or Plasmax, respectively. Data are represented as mean ± SD of triplicate wells. **H** Intracellular levels of dTTP measured by LC–MS after 4 h incubation with 400 µM or 140 µM of 2,3,3-D_3_-Serine in DMEM or Plasmax, respectively. Data are represented as mean ± SD of triplicate wells. **I** Intracellular levels of AMP and ATP measured by LC–MS after 4 h incubation with 400 µM or 140 µM of 2,3,3-D_3_-serine in DMEM or Plasmax, respectively. Data are represented as mean ± SD of triplicate wells. **J** Intracellular levels of ATP measured by LC–MS after 4 h incubation with 400 µM or 330 µM of 1-^13^C-glycine in DMEM or Plasmax, respectively. Data are represented as mean ± SD of triplicate wells. All graphs are representative of 3 independent experiments. Source data are provided as a Source Data file.

We examined three cancer cell lines from different cancer types, A549 (lung cancer), HCT116 (colon cancer) and MDA-MB-231 (breast cancer), whose proliferation is highly dependent on exogenous serine and glycine when grown in DMEM (Fig. 1A)^3,4^. The proliferation rate of each of these cell lines was similar in DMEM or Plasmax even though Plasmax contains 4-fold less FBS than DMEM (2.5% for Plasmax and 10% for DMEM) (Supplementary Fig. 1A). However, cells in Plasmax were clearly less sensitive to serine/glycine depletion (Fig. 1A). All the proliferation assays were carried out in media containing non-dialysed serum (standard for these assays), which provides low levels of some nutrients, including serine. However, since Plasmax contains less serum than DMEM, this residual serine cannot account for the difference in proliferation. For comparison, MDA-MB-468 (breast cancer) cells that carry a PHGDH amplification^10^ remained resistant to serine/glycine restriction in DMEM and Plasmax (Supplementary Fig. 1B). Consistent with previous observation, extracellular serine was less rapidly taken up from Plasmax compared to DMEM without any compensatory increase in glycine uptake (Supplementary Fig. 1C, D)^16^. Furthermore, increasing the serine and glycine levels in Plasmax (140 µM and 330 µM, respectively) to those found in DMEM (400 µM) did not enhance the rate of serine uptake of A549 cells (Supplementary Fig. 1E). Plasmax and DMEM contain different concentrations of several other amino acids (Supplementary Fig. 1F), including alanine, proline, glutamate, asparagine and aspartate which are not present in DMEM. We therefore considered whether any of these differences might influence the ability of cells to import serine. Serine is a neutral amino acid that can be transported into the cell through the sodium-dependent transporter ASC (ASCT1 and ASCT2), the sodium-dependent transporter system A (SNAT1 and SNAT2) or the sodium-independent transporter (ASC1)^20^. These transporters are also able to shuttle other neutral amino acids such as alanine, which is present in Plasmax (500 µM) but not in DMEM (0 µM)^21^. Alanine has been shown to inhibit L-serine transport by ASCT1 and ASCT2 when expressed in HEK cells^22^ and we noted that *ASCT1 (SLC1A4)* and *ASCT2 (SLC1A5)* transcription is upregulated in cells grown in Plasmax (Supplementary Fig. 1G). Removal of alanine from Plasmax increased intracellular levels of labelled serine and decreased the levels of labelled serine in the supernatant of HCT116, A549 and MDA-MB-468 (Supplementary Fig. 1H, I), while supplementing DMEM with alanine reduced the intracellular levels of serine in A549 cells (Supplementary Fig. 1J). Taken together, these data suggest that the composition of the media can influence the transport of amino acids, such as serine.

The reduced serine uptake in cells cultured in Plasmax resulted in lower intracellular serine levels (Fig. 1B), prompting us to consider whether the utilisation of serine in these cells differed from cells grown in DMEM. Serine is used in numerous metabolic pathways, providing glycine and one-carbon units for lipid and nucleotide synthesis and also maintaining the redox balance through glutathione and NADPH synthesis (Fig. 1C)^23^. When serine is plentiful, the flux through the one-carbon cycle exceeds requirements for nucleotide synthesis and results in formate overflow^24^. Cells cultured in Plasmax (for metabolomics and NMR studies dialysed serum was used to avoid confounding contributions from serum metabolites) consistently showed significantly reduced formate overflow as measured by NMR, suggesting a lower rate of one-carbon metabolism (Fig. 1D). Using deuterium-labelled serine (2,3,3-D_3_-Serine), we found that there is a lower contribution of extracellular serine to the intracellular pool of glycine in cells grown in Plasmax, as shown by reduced levels of M + 1 glycine (Fig. 1E and Supplementary Fig. 1K). The extracellular serine contribution to the glutathione (GSH) pool in Plasmax was also slightly reduced (Fig. 1F) while cells increased the use of exogenous glycine to maintain the levels of GSH, as observed by higher labelling from glycine into M + 2 GSH in Plasmax using deuterium labelled glycine (2,2-D_2_ Glycine) (Fig. 1G and Supplementary Fig. 1L). Overall, GSH levels appeared to be protected in cells grown in Plasmax. Furthermore, there was a clear reduction in exogenous serine contribution to de novo purine and thymidylate synthesis, indicated by a lower accumulation of M + 1 dTTP and M + 2 AMP and ATP in cells grown in the presence of deuterium-labelled serine (2,3,3-D_3_ Serine) (Fig. 1H, I) accompanied by a small drop in total thymidylate (in MDA-MB-468 and HCT116 cells) and total AMP and ATP (MDA-MB-468 and A549 cells). Interestingly, these cells also show less ATP derived from carbon-labelled glycine (Fig. 1J), suggesting a reduction in de novo purine synthesis from both extracellular serine and glycine. These data suggest that cells grown in Plasmax accommodate to decreased serine uptake by reducing one-carbon flux—thereby lowering the excess production of formate—and channelling exogenous glycine to maintain GSH levels (Fig. 1G). The decrease in use of extracellular serine and glycine for purine and thymidylate synthesis does not, however, strongly impact overall levels of these nucleotides and proliferation rates are maintained.

### Upregulation of the de novo serine synthesis pathway in Plasmax

**Fig. 2:**
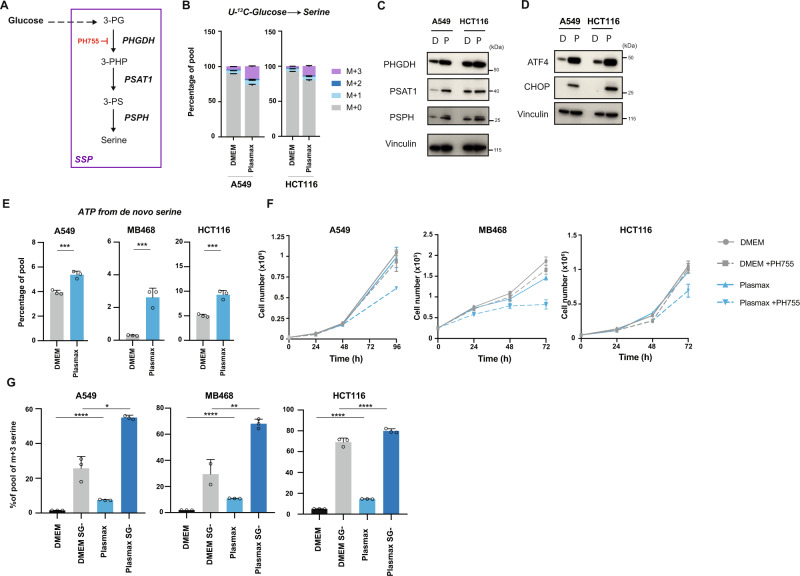
Upregulation of the de novo serine synthesis pathway in Plasmax. **A** Serine can be synthesised de novo from 3-phosphoglycerate (3-PG) derived from glucose by a 3-step enzymatic reaction. The first is an NAD^+^-dependent reaction catalysed by PHGDH which converts 3-PG into 3-phosphohydroxypyruvate (3-PHP). 3-PHP is then converted by PSAT1 into 3-phosphoserine (3-PS) which is subsequently hydrolysed into serine by PSPH. **B** Percentage of pool of intracellular serine measured by LC–MS after incubation for 4 h with 22.5 mM or 5.5 mM of U-^13^C-glucose in DMEM or Plasmax, respectively. Data are represented as mean ± SD of triplicate wells. **C** Western blot of SSP enzymes of cells grown in DMEM (D) or Plasmax (P) for 72 h. Membrane was reprobed with Vinculin as a loading control. Blots are representative of 2 independent experiments. **D** Western blot of ATF-4 pathway of cells grown in DMEM (D) or Plasmax (P) for 72 h. Membrane was reprobed with Vinculin as a loading control. Blots are representative of 3 independent experiments. **E** Sum of ATP M + 6, ATP M + 7, ATP M + 8, ATP M + 9 percentage of pools measured by LC–MS after incubation for 4 h with 22.5 mM or 5.5 mM of U-^13^C-glucose in DMEM (grey) or Plasmax (blue). Data are represented as mean ± SD of triplicate wells (unpaired Student’s *t* test, ***p < 0.001). **F** Growth curve of cells grown in DMEM (grey) or Plasmax (blue) with or without 10 µM of PH755. Data are represented as mean ± SD of triplicate wells. **G** Percentage of M + 3 serine pools after incubation for 4 h with U-^13^C-glucose in DMEM (black), DMEM SG- (grey), Plasmax (light blue) or Plasmax SG- (dark blue) with or without serine/glycine (SG-). Data are represented as mean ± SD of triplicate wells (multiple comparison by one-way Anova, *p < 0.05, **p < 0.01, ***p < 0.001, ****p < 0.0001). All graphs are a representative of 3 independent experiments. Source data are provided as a Source Data file.

Our observations that cells cultured in Plasmax generated lower amounts of purines from exogenous serine led us to examine the possibility that these cells increase de novo serine synthesis. The serine synthesis pathway (SSP) starts with the glycolytic intermediate, 3-phosphoglycerate (3-PG), which is oxidised to 3-phosphohydroxypyruvate (3-PHP) by PHGDH, followed by two further reactions catalysed by phosphoserine aminotransferase (PSAT1) and phosphoserine phosphatase (PSPH) to produce serine (Fig. 2A). This pathway is upregulated in serine-depleted conditions^25,26^ and in some tumours, including a subset of colorectal, breast and non-small cell lung (NSCL) cancers^10,27,28^. Using A549 and HCT116, we found an increase in the percentage of M + 3 serine and M + 2 glycine derived from fully labelled glucose (U-^13^C-Glucose) in cells cultured in Plasmax (5.5 mM glucose) compared to DMEM (22.5 mM glucose) (Fig. 2B and Supplementary Fig. 2A). Increasing the serine and glycine concentration in Plasmax to that present in DMEM did not prevent this increase in glucose-derived serine in either cell line (Supplementary Fig. 2B). While Plasmax has lower levels of glucose than DMEM, increasing the glucose concentration in Plasmax or decreasing them in DMEM did not lead to a change in the pool of M + 3 serine (Supplementary Fig. 2C). The enhanced de novo serine synthesis in Plasmax was accompanied by elevated expression of all three SSP enzymes, PHGDH, PSAT1 and PSPH (Fig. 2C). Each of the SSP enzymes can be induced through activating transcription factor-4 (ATF-4), which is typically upregulated under nutrient depletion^26^. Indeed, we observed increased ATF-4 protein expression in cells cultured in Plasmax as well as induction of CHOP, another downstream target of ATF-4 (Fig. 2D). This finding led us to investigate the intracellular availability of nutrients in cells grown in these conditions. The ratios between intracellular amino acid levels in Plasmax and DMEM were broadly consistent with the ratios of extracellular amino acids levels (Supplementary Figs. 1F and 2D), with a lower level of several amino acids in Plasmax. Modulation of numerous amino acids including arginine—which is considerably lower in Plasmax (Plasmax contains 64 µM of arginine compared to 400 µM in DMEM)—can trigger an ATF-4 response^29,30^. We first examined the effect of restoring the levels of several amino acids that are lower in plasmax compared to DMEM (LEU, ILE, MET, GLN, ARG). Only the supplementation of arginine in Plasmax to the level used in DMEM reduced ATF-4 and PHGDH expression (Supplementary Fig. 2E, F) while reducing arginine in DMEM to levels present in Plasmax, induced ATF4 and PHGDH expression (Supplementary Fig. 2G). However, increasing the serine levels in Plasmax to 400 µM (as in DMEM) did not change the expression of ATF-4 or PHGDH (Supplementary Fig. 2F). These results indicated that the super-physiological levels of amino acids such as arginine in DMEM suppress a level of ATF-4 activity that is seen under more physiological concentrations of these amino acids, which is in line with upregulation of the ATF-4 pathway in human and mouse tumour compared to normal tissue^31^.

Confirming the increased contribution of the SSP in cells grown in Plasmax compared to DMEM, we noted an increase in the pools of M + 6, M + 7, M + 8, M + 9 labelled ATP, which appear when U-^13^C-glucose-derived glycine and one-carbon units are used to synthesise purines in cells fed carbon-labelled glucose (Fig. 2E). To assess the contribution of this increase in de novo serine synthesis in Plasmax to proliferation, we examined the effect of treating cells with the PHGDH inhibitor, PH755^32^. While PHGDH inhibition had no effect on cancer cells growth in DMEM, it significantly reduced the proliferation of cells cultured in Plasmax (Fig. 2F). These data suggest that cells cultured in Plasmax are more dependent on de novo synthesised serine as a source of one-carbon units and to support purine synthesis and proliferation. However, the total pool of intracellular serine does not significantly change after treatment with PH755 indicating that cells can revert to serine uptake during PHGDH inhibition (Supplementary Fig. 2H). This was confirmed by the rescue of the anti-proliferative effect of PH755 by supplementing the cells with excess serine (Supplementary Fig. 2I). In addition, while removal of serine and glycine from either DMEM or Plasmax led to an increase in de novo serine synthesis, this response was more robust in Plasmax, where this pathway was already induced in the presence of exogenous serine (Fig. 2G). It would appear that amino acid availability increases SSP activity in Plasmax, allowing cells to be less sensitive to the removal of exogenous serine.

### Hypoxanthine salvage is a major contributor of purines in Plasmax

We next considered that other differences between DMEM and Plasmax could impact the dependence on exogenous serine. Previous studies showed that formate, which is present at 33 µM in Plasmax but absent from DMEM, can provide one-carbon units to support purine synthesis and—in cooperation with glycine—proliferation^4^. Tracing ^13^C-labelled formate into ATP in both Plasmax and DMEM showed that cells grown in either media could access exogenous formate for purine synthesis (Supplementary Fig. 3A). However, depleting formate from Plasmax did not lower the overall levels of ATP or lead to an increased incorporation of serine-derived nitrogen and carbons into ATP (Supplementary Fig. 3B), indicating that while the free formate in Plasmax could contribute to ATP synthesis, it does not influence the contribution of extracellular serine to de novo purine synthesis (Supplementary Fig. 3B).

**Fig. 3:**
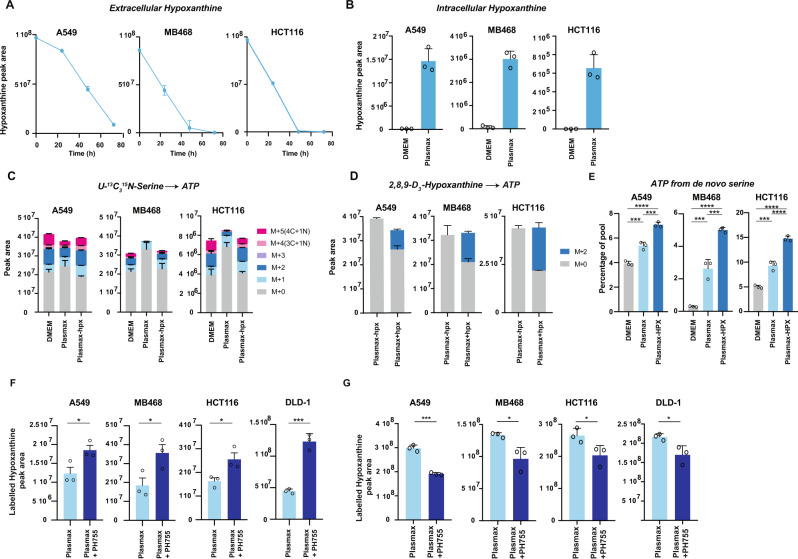
Hypoxanthine salvage is a major contributor of purines in Plasmax. **A** Extracellular levels of hypoxanthine measured by LC–MS in supernatants of cells grown in Plasmax (blue) for up to 72 h. Data are represented as mean ± SD of triplicate wells. **B** Intracellular levels of hypoxanthine measured by LC–MS in cells grown in DMEM (grey) or Plasmax (Blue). Data are represented as mean ± SD of triplicate wells. **C** Intracellular levels of ATP measured by LC–MS after incubation for 4 h with 400, 140 or 140 µM of ^13^C_3_-^15^N-Serine in DMEM, Plasmax or Plasmax-hpx, respectively. Data are represented as mean ± SD of triplicate wells. **D** Intracellular levels of ATP measured by LC–MS after incubation for 4 h with 5 µM of 2,8,9-D_3_-hypoxanthine or not in Plasmax. Data are represented as mean ± SD of triplicate wells. **E** Sum of ATP M + 6, ATP M + 7, ATP M + 8, ATP M + 9 pools measured by LC–MS after incubation of cells for 4 h with 22.5 mM or 5.5 mM U-^13^C-glucose in DMEM (grey), Plasmax (light blue) or,Plasmax-hpx (dark blue), respectively (same experiment as Fig. 2F but with an extra condition). Data are represented as mean ± SD of triplicate wells (one-way Anova, ***p < 0.001, ****p < 0.0001). **F** Cells were grown 24 h in Plasmax containing 2,8,9-D_3_-hypoxanthine with (light blue) or without (dark blue) 10 µM PH755 before intracellular labelled 2,8,9-D_3_-hypoxanthine was measured by LC–MS. Data are represented as mean ± SD of triplicate wells (unpaired Student’s *t* test, *p < 0.05, ***p < 0.001). **G** Cells were grown 24 h in Plasmax containing 2,8,9-D_3_-hypoxanthine with (light blue) or without (dark blue) 10 µM PH755 before labelled 2,8,9-D_3_-hypoxanthine was measured in supernatants by LC–MS. Data are represented as mean ± SD of triplicate wells (unpaired Student’s *t* test, *p < 0.05, ***p < 0.001). All graphs are representative of 3 independent experiments. Source data are provided as a Source Data file.

In addition to de novo synthesis, purines can be synthesised through the salvage pathway, which recycles the hypoxanthine derived from degraded bases and transfers it to a phosphoribosyl group to form inosine monophosphate (IMP), so maintaining the levels of adenylates^33,34^. Hypoxanthine is not present in DMEM but Plasmax contains 5 µM hypoxanthine, reflecting the level in human circulation. Cells grown in Plasmax rapidly deplete hypoxanthine from the extracellular media and accumulate high intracellular levels compared to cells grown in DMEM (Fig. 3A, B). To determine whether the lower uptake of exogenous serine seen in cells grown in Plasmax is the result of hypoxanthine utilisation, we removed hypoxanthine from the Plasmax and looked at incorporation of fully labelled ^13^C_3_-^15^N-serine into ATP. As observed in Fig. 1I, exogenous serine incorporation into ATP was lower in the three cells lines cultured in Plasmax compared to DMEM (Fig. 3C). However, removal of hypoxanthine from Plasmax fully restored the levels of exogenous serine utilisation to those seen in DMEM (Fig. 3C). Furthermore, addition of hypoxanthine to DMEM significantly reduced the incorporation of serine into ATP, confirming that cancer cells preferentially salvage hypoxanthine instead of synthesising de novo ATP from extracellular serine (Supplementary Fig. 3C). Indeed, by tracing deuterium-labelled hypoxanthine we evaluated its direct contribution to the ATP pool, which consistently ranged from 20 to 40% in both DMEM and Plasmax (Fig. 3D and Supplementary Fig. 3D). This proportion of ATP derived from hypoxanthine is generally higher than the pools of ATP synthesised from media-derived or de novo synthesised serine (Supplementary Fig. 3E). Furthermore, removal of hypoxanthine from Plasmax increased the proportions of de novo synthesised serine labelling of ATP, along with an increase in ATP derived from exogenous serine (Fig. 3E and Supplementary Fig. 3E). Taken together, these data suggest that both de novo serine and hypoxanthine contribute to the pools of ATP in cells cultured in Plasmax. Our findings corroborate different studies in human cancer patients that suggest that cancer cells do not exclusively synthetise purines from extracellular serine but partially from hypoxanthine^35–37^. In the cell lines that we have tested, hypoxanthine salvage seems to be preferentially used to provide purines instead of de novo synthesis from serine.

Next, we aimed to determine if the presence of hypoxanthine was able to mitigate the anti-proliferative effect of serine and glycine deprivation in physiological conditions. To assess this in vitro and in vivo, we included DLD-1, a colorectal cancer cell line resistant to serine/glycine starvation in DMEM and which we have previously studied in vivo^9^. When cultured in Plasmax, we observed that DLD-1 cells showed enhanced de novo serine synthesis, became sensitive to PHGDH inhibition with PH755 and had a reduced contribution of serine to de novo purine synthesis due to the presence of hypoxanthine, similar to the other cell lines (Supplementary Fig. 3F–H).

### Cooperation between serine limitation and modulation of hypoxanthine in limiting tumour cell growth

**Fig. 4:**
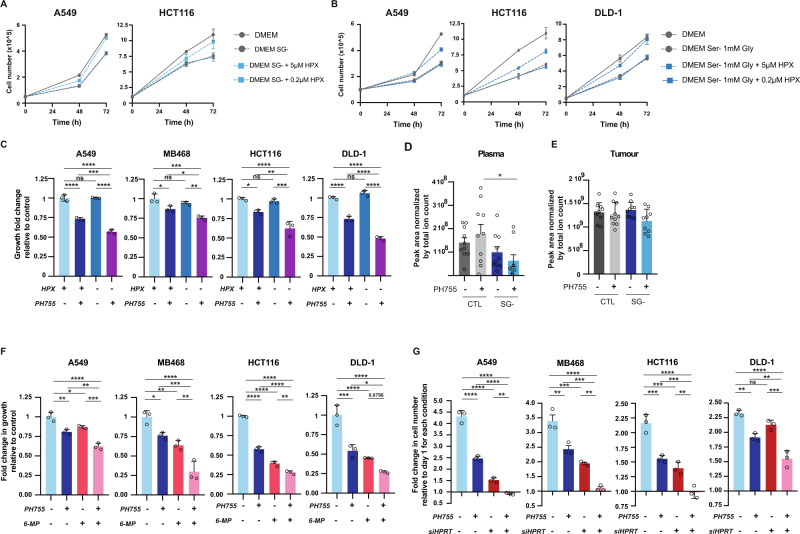
Cooperation between serine synthesis pathway inhibition and modulation of hypoxanthine in limiting tumour cell growth. **A** Growth curve of cells grown in DMEM lacking serine and glycine (-SG) supplemented (blue) or not (grey) with 5 or 0.2 µM of hypoxanthine. Data are represented as mean ± SD of triplicate wells and representative of 3 independent experiments. **B** Growth curve of cells grown in DMEM lacking serine (−S) but containing 1 mM glycine (+1 mM G) supplemented (blue) or not (grey) with 5 or 0.2 µM of hypoxanthine. Data are represented as mean ± SD of triplicate wells and representative of 3 independent experiments. **C** Growth fold change relative to the untreated Plasmax condition (HPX+ /PH755−) after 72 h of proliferation in the different conditions. Cells were treated with 10 µM PH755 where indicated. Data are represented as mean ± SD of triplicate wells and representative of three independent experiments (one-way Anova, *p < 0.05, **p < 0.01, ***p < 0.001, ****p < 0.0001, ns: no significance). **D**, **E** Levels of hypoxanthine measured by LC–MS in plasma and tumour, respectively, collected at end-point from DLD-1 xenograft-bearing mice fed with control diet and treated with vehicle *n* = 10 (dark grey) or PH755 *n* = 10 (grey) or serine/glycine free diet and treated with vehicle (light blue) *n* = 10 or PH755 *n* = 9 (dark blue). Levels of hypoxanthine were normalised by total ion count. Data are represented as mean ± SEM (multiple comparison by one-way Anova, *p < 0.05). The samples were obtained from an experiment previously published^9^. **F** Fold change in growth relative to the untreated Plasmax condition (PH755-/6-MP-) after 72 h of proliferation in the different conditions. Cells were all treated with 10 µM PH755 where indicated and with 1 µM 6-MP for MDA-MB-468, 2.5 µM 6-MP for A549 and HCT116 and 5 µM 6-MP for DLD-1 when indicated. PH755-/6-MP- (light blue), PH755+ /6-MP− (dark blue), PH755−/6-MP+ (red), PH755+ /6-MP+ (pink). Data are represented as mean ± SD of triplicate wells and representative of three independent experiments (multiple comparison by one-way Anova, *p < 0.05, **p < 0.01, ***p < 0.001, ****p < 0.0001). **G** Cells were transfected at day 0 with siHPRT1 or non-targeting siRNA. At day 1, cells were transferred into Plasmax media with or without 10 µM PH755 Graphs represent fold change of cell number after 72 h relative to day 1. PH755-/siHPRT- (light blue), PH755+ /siHPRT− (dark blue), PH755−/siHPRT+ (red), PH755+ /siHPRT+ (pink). Data are represented as mean ± SD of triplicate wells and representative of two independent experiments (one-way Anova, **p < 0.01, ***p < 0.001, ****p < 0.0001, ns no significance). Source data are provided as a Source Data file.

To test the contribution of hypoxanthine to purine pools when serine availability is limited, we first measured intracellular levels of labelled hypoxanthine when cells were grown in Plasmax and treated with the PHGDH inhibitor or serine and glycine deprivation. We observed increased levels of intracellular hypoxanthine both after serine/glycine starvation and PHGDH inhibition (Fig. 3F and Supplementary Fig. 3I). Extracellular levels of hypoxanthine were also depleted after treatment with PH755 (Fig. 3G). In addition, even though hypoxanthine supplementation in full medium did not affect cell proliferation (Supplementary Fig. 4A), hypoxanthine supplementation rescued proliferation of cells in serine/glycine-free DMEM, presumably by allowing endogenously produced serine and glycine to be used in processes other than de novo purine synthesis (Fig. 4A). The hypoxanthine also partially rescued proliferation in serine-starved cells supplemented with glycine, similar to the effect of formate (Fig. 4B)^38^ and supporting the proposal that cells use hypoxanthine to maintain ATP levels. Cell proliferation in serine/glycine-free Plasmax depleted of hypoxanthine was only modestly reduced (Supplementary Fig. 4B). However, combination of PH755 with a lack of hypoxanthine resulted in further reduction of cancer cell proliferation compared to treatment of PH755 alone (Fig. 4C). These results support the increased dependence of cells in Plasmax on endogenous serine synthesis and indicate that the increased hypoxanthine uptake in response to PHGDH inhibition can support cell proliferation.

Tumours are irrigated by the vasculature, which provides the interstitial space with a nutrient rich fluid termed the interstitial fluid (TIF)^39^. As shown previously, we found that circulating hypoxanthine levels in mice (0.02–0.2 µM) were lower than those in humans (∼5 µM)^17^. Furthermore, TIF from tumours formed from DLD-1 cells—which utilised hypoxanthine in vitro—also showed a similarly low range of hypoxanthine levels (∼0.18 µM for plasma and ∼0.77 µM for TIF (Supplementary Fig. 4C). To determine the impact of serine/glycine limitation on circulating and tumour levels of hypoxanthine, we analysed serum and tumours from mice carrying DLD-1 xenograft tumours and treated with a serine/glycine-free diet in combination with the PHGDH inhibitor, PH755. These samples were generated for a previous study that showed an inhibition of tumour growth in the double treated mice^9^. Although there was considerable variability between samples, we observed a trend towards reduction in circulating hypoxanthine levels (Fig. 4D) that paralleled the reduction in circulating serine and glycine levels (Supplementary Fig. 4D). Interestingly, tumoral levels of hypoxanthine did not decrease, consistent with an ability of tumour cells to take up exogenous hypoxanthine to support nucleotide synthesis under serine limiting conditions (Fig. 4E).

Our work predicts that limiting hypoxanthine metabolism would cooperate with limitation of serine availability to inhibit the growth of cancer cells in patients. This may be difficult to test in mice, where the concentration of circulating hypoxanthine (0.2 uM) was shown to be insufficient to improve the proliferation of serine/glycine starved cells (Fig. 4A, B). We therefore looked for a potential cooperation between serine starvation and hypoxanthine limitation in Plasmax. Initially we treated cells with 6-mercaptopurine (6-MP), an anti-metabolite that competes with hypoxanthine for hypoxanthine-guanine phosphoribosyltransferase (HPRT), the enzyme required to utilise hypoxanthine for purine synthesis. HPRT activity also leads to the conversion of 6-MP into 5-thio-ionsinic acid (TIMP), which inhibits the conversion of IMP to AMP. Upon treatment with 6-MP for 2 h we detected the expected production of TIMP (Supplementary Fig. 4E) and some inhibition of proliferation after 72 h (Fig. 4F) although this treatment did not affect the proliferation of cells in DMEM (Supplementary Fig. 4F). The combination of 6-MP and PH755 resulted in a significantly greater decrease in proliferation compared to each single treatment in 3 out of 4 cell lines (Fig. 4F).

To assess directly the effect of a reduction in HPRT activity, we used siRNA to deplete cells of HPRT expression (Supplementary Fig. 4G). Knockdown of HPRT led to a reduction of cell growth in Plasmax confirming the importance of this pathway in physiological medium (Fig. 4G). Interestingly, although removal of hypoxanthine from Plasmax leads to increased purine production from both de novo synthesised serine and exogenous serine (Supplementary Fig. 3E), the combination of HPRT depletion and PHGDH inhibition resulted in complete abrogation of cell proliferation (Fig. 4G). Since these cells show enhanced serine uptake, these results suggest additional consequences of HPRT depletion leading to proliferative arrest. Taken together, these observations show that the resistance of cells in Plasmax to serine and glycine deprivation is due to a combination of mechanisms reflecting the rewiring of the serine and one-carbon metabolism in this culture medium.

## Discussion

Our data indicate that the contribution of de novo serine synthesis in cancer cells to the nucleotide pool is underestimated when cells are cultured in classical growth media—in which a greater dependency on exogenous serine is seen. Using Plasmax to more accurately represent metabolite levels available in human plasma, we noted a higher basal level of SSP activity compared to classical media reflected in elevated ATF-4 expression, potentially reflecting levels of amino-acids such as arginine. The higher SSP enzyme expression and activity that we observe in physiological media is in line with the increasing evidence that SSP enzymes are highly important in cancer biology^6,40,41^. This study also demonstrates the contribution that hypoxanthine provides to support purine synthesis and reduce the dependence on exogenous serine and glycine. Previous studies have suggested that neoplastic cells initiate increased purine salvage to gain a growth advantage^36^, with elevation of the expression of HPRT, a key enzyme in the salvage pathway, in 50% of lung carcinoma patients^42^. Although hypoxanthine was reduced in the plasma of tumour bearing mice fed with a serine/glycine-deprived chow and treated with a de novo serine synthesis inhibitor, maintenance of hypoxanthine levels in the tumour suggest that this metabolite is available and utilised by the tumour when serine is limiting. While dietary strategies to limit circulating serine and glycine have been shown to be therapeutically effective in a number of mouse cancer models, this work suggests that targeting de novo serine synthesis and hypoxanthine availability may significantly enhance the response to circulating serine and glycine limitation. Dietary modulation of hypoxanthine has been described^43^ and the targeting of hypoxanthine transporters can affect tumour proliferation^44^. Our study highlights the potential of physiological media to reveal metabolic vulnerabilities of cancer cells that may provide additional therapeutic targets.

## Methods

### Cell culture

All cell lines were obtained from the Francis Crick Institute Cell Services Science Technology Platform, which ensures various quality controls such as mycoplasma testing, STR profiling and species validation. All cell lines were maintained in DMEM (Gibco, 41966) supplemented with 10% FBS and penicillin–streptomycin at 37 °C in humidified atmosphere of 5% CO_2_. For experimental work, cells were transferred to test media 24 h prior to the start of the experiments. Test media was formulated with MEM (Gibco) supplemented with 2 mM L-glutamine, 17 mM D-glucose (to a final concentration of 22.5 mM glucose) and MEM vitamins (SG- medium) while control media was further supplemented with 400 µM L-serine and glycine (termed DMEM). Plasmax and all its formulation in the manuscript were made in the lab on the basis of the formulation previously published^16^.

### Proliferation assay

For all proliferation assays non-dialysed FBS was used at 10% in DMEM and 2.5% in Plasmax. For Fig. 1A, Supplementary Fig. 1A and Supplementary Fig. 1B all cell lines were plated at 2 × 10^4^ cells in 6-well plates in DMEM with 3 replicates per time point. After 24 h cells were washed with PBS and transferred to the experimental conditions. Cells were then trypsinized, diluted in PBS-EDTA and counted with a CASY Model TT Cell Counter (Innovatis, Roche Applied Science).

For Fig. 2F and Supplementary Fig. 3F, A549, HCT116, MDA-MB-468 and DLD-1 cells were plated at 2.5 × 10^4^ cells in a 12-well plate in DMEM with three replicates per time point and condition. After 24 h of acclimatisation in Plasmax or DMEM cells were washed with PBS and transferred to the experimental conditions and treated with 10 µM PH755 (Raze Therapeutics) diluted in DMSO or DMSO alone. Media were changed after 48 h and cells were counted as described above.

For Supplementary Fig. 2I, HCT116, A549 and MDA-MB-468 were plated at 2.5 × 10^4^ cells in a 12-well plate in Plasmax with three replicates per condition. After 24 h of acclimatisation in Plasmax cells were washed with PBS and transferred to the different experimental conditions. Where indicated cells were treated with 10 µM PH755 (Raze Therapeutics) diluted in DMSO or DMSO alone. For serine supplementation, cells in Plasmax received an additional 400 µM diluted in PBS. Media were changed after 48 h and cells were counted as described above. Relative cell number was calculated to the number of cells after 72 h in the complete Plasmax media condition.

In Supplementary Fig. 4A, A549 and HCT116 cells were plated at 5 × 10^4^ cells in a 12-well plate in DMEM with three replicates per time point and condition. In Fig. 4A, B, A549, HCT116 and DLD-1 cells were plated at 5 × 10^4^ in a 12-well plate in DMEM with three replicates per time point and condition. After 24 h of acclimatisation in Plasmax or DMEM cells in indicated media was supplemented with 5 µM or 0.2 µM hypoxanthine diluted in DMSO or DMSO alone from time point 0. Cell counting was performed as described above. Media were changed after 48 h and cells were counted as described above.

For Fig. 4C and Supplementary Fig. 4B, HCT116, A549, MDA-MB-468 and DLD-1 were plated at 2.5 × 10^4^ cells in a 12-well plate in Plasmax with three replicates per condition. After 24 h of acclimatisation in Plasmax cells were washed with PBS and transferred to the different experimental conditions. For Fig. 4C cells received 10 µM of PH755 (Raze Therapeutics) diluted in DMSO or DMSO alone when indicated. Media were changed after 48 h and cells were counted as described above after 72 h. Relative cell number was calculated to the number of cells after 72 h in the complete Plasmax media condition.

For Fig. 4F, HCT116, A549, MDA-MB-468 and DLD-1 were plated at 2.5 × 10^4^ cells in a 12-well plate in Plasmax with three replicates per condition. After 24 h of acclimatisation in Plasmax cells were washed with PBS and transferred to the different experimental conditions. When indicated cells received 10 µM of PH755 (Raze Therapeutics) diluted in DMSO or DMSO alone. For 6-Mercaptopurine (6-MP) (Merck) MDA-MB-468 cells received 1 µM, A549 and HCT116 received 2.5 µM and DLD-1 received 5 µM diluted in DMSO when indicated. Media were changed after 48 h and cells were counted as described above after 72 h. Relative cell number was calculated to the number of cells after 72 h in the complete Plasmax media condition.

For Fig. 4G, HCT116, A549, MDA-MB-468 and DLD-1 were plated at 2.5 × 10^4^ cells in a 12-well plate and transfected with a pool of siRNA targeting HPRT (D-008735-01) or a non-targeting siRNA (D-001206-13) from Dharmacon. After 24 h, media were changed to the treatment media. When indicated cells received 10 µM of PH755 (Raze Therapeutics) diluted in DMSO or DMSO alone. Media were changed after 48 h and cells were counted at 72 h. Relative cell number was calculated to the number of cells when media were changed to treatment media.

### Western blot

After pelleting, cells were lysed with RIPA-buffer (Millipore) supplemented with phosphatase inhibitor cocktail (ThermoFisher Scientific) and complete protease inhibitors (Roche). Proteins were separated using precast 4–12% bis-tris gels (Invitrogen) and transferred to nitrocellulose by dry-transfer (iBlot, Thermofisher Scientific). Membranes were probed with the following primary antibodies: PHGDH (#13428), ATF4 (#11815) and CHOP (#2895) from Cell Signalling Technology; PSAT (ab96136), PSPH (ab96414) and HPRT (ab133242), Actin (ab8226) from Abcam; Vinculin (sc-73614) from Santa Cruz Biotechnology. All antibodies were used at a dilution of 1:1000. Blot were developed with ECL chemiluminescence kits (Pierce) after incubation with appropriate species-specific horseradish peroxidase-conjugated antibodies. All antibodies were verified and confirmed for species as per the manufacturers’ disclosure. Additional validation was performed for the PHGDH antibody using a PHGDH KO cell line. PSAT and PSPH antibodies were validated under serine/glycine free conditions.

### Liquid chromatography–mass spectrometry

For all LC–MS experiments dialysed FBS was used for DMEM (10%) or Plasmax (2.5%).

For metabolites in supernatants, 2 × 10^5^ of HCT116, A549 and MDA-MB-468 were plated in 6-well plates in DMEM for 24 h in order to attach. Cells were then switched to fresh DMEM, Plasmax, Plasmax (400 µM SER, 400 µM GLY) or Plasmax without alanine and kept for up to 96 h. 10 µl of supernatant of each time point was extracted with 490 µL of ice-cold extraction buffer (methanol/acetonitrile/water in the 50/30/20 ratio), vortexed and spun at 15,000 *g* for 12 min at 4 °C. Supernatants were used for LC–MS analysis.

For labelling experiments, 2 × 10^5^ of HCT116, A549, MDA-MB-468 and DLD-1 cells were plated in 6-well plates in the experimental media 24 h prior to the labelling. Duplicate plates were used for cell counting in order to normalise the LC–MS analysis on the basis of cell number. After 24 h in the experimental media, media were replaced by the equivalent media depleted of the metabolite of interest and supplemented with the same amount of the labelled metabolite for 4 h. In the case of treatment the compound was added at the same time as the labelling media. Labelled compounds used were: ^13^C_3_-^15^N-serine (#608130) and 2,2-D_2_-glycine (#336459) from Sigma-Aldrich; U-^13^C-glucose (CLM-1396), 1-^13^C-glycine (CLM-422), 2,3,3-D_3_-serine (DLM-582), 2,8,9-D_3_-hypoxanthine (DLM-2923) from Cambridge Isotopes. Cells were washed with PBS and metabolites were extracted in the plate with the same extraction buffer as described above. After vortexing and centrifugation at 15,000 *g* for 12 min at 4 °C, supernatants were used for LC–MS analysis.

For the analysis of tumour samples, tissue (20–40 mg/mL of described extraction buffer) was homogenised in a precellys 24 homogeniser (Bertin Instruments). Homogenate was collected and spun at 15,000 *g* for 12 min at 4 °C and the supernatant collected for LC–MS analysis.

For plasma samples, plasma was diluted 20-fold with the same extraction buffer, vortexted and spun at 15,000 *g* for 12 min at 4 °C. The supernatant was collected for analysis.

For tumour interstitial fluid (TIF), extracted tumours were placed in Spin-x filter tubes (Costar 8170) and centrifuged sequentially at 100 *g*, 400 *g* and 1500 *g* for 10 min at 4 °C. TIF was the diluted 20-fold in the same extraction buffer, vortexed and spun at 15,000 *g* for 12 min at 4 °C. Supernatant was collected for analysis. LC–MS analysis was performed as previously described^4^. Data were analysed with TraceFinder and Xcalibur from Thermofisher.

### NMR

NMR was used to measure formate as represented in Fig. 1D. HCT116, A549 and MDA-MB-468 were plated at 1 × 10^5^ in 6 well plates and left for three days in DMEM or Plasmax. 160 µL of supernatant was collected for NMR analysis and cells were counted as already described to allow normalisation to cell number. A solution of 10 mM DSS (sodium 2,2-dimethyl-2-silapentane-5-sulfonate from Sigma) diluted in D20 was added to the samples to obtain a final concentration of 0.1 mM DSS. NMR spectra were acquired at 25 °C with a Bruker Avance III HD instrument with a nominal ^1^H frequency of 700 MHz using 3 mm tubes in a 5 mm CPTCI cryoprobe. For ^1^H 1D profiling spectra the Bruker pulse program *zgesgppe* for excitation sculpting with pure echo^45^ was used with 20 ppm sweep width, 1 s relaxation delay and 4 s acquisition time. Typically, 128 transients were acquired. Data were processed and analysed using *Chenomx NMR Suite* (Chenomx, Edmonton, Canada). Free induction decays were zero-filled, apodized with exponential multiplication (line-broadening factor LB = 1 Hz), Fourier-transformed and the resulting spectra were then phase corrected before baseline correction, all in the Processor component of the Chenomx software. Formate quantitation was performed based on the chemical shift reference (DSS) assumed to be at 0.1 mM concentration and with linewidth adjusted to obtain a good fit to the Chenomx library spectra for multiple metabolites in the spectrum.

### RT-qPCR

Total RNA was extracted using TriPure isolation reagent (Roche) from at least two technical replicates. cDNA was generated using the high-capacity cDNA reverse transcription kit (ThermoFisher) according to manufacturer’s instructions. IDT PrimeTime^®^ gene expression master mix (IDT) was used to perform qPCR with the following predesigned primer/probe assay from IDT; *ASCT1* (Hs.PT.58.4102571), *ASCT2* (Hs.PT.58.21358468), *POLRA* (Hs.PT.39a.19639531). Sequences can be found in Supplementary Table 1. Data were collected on a QuantStudio 3 system from applied biosystems.

### In vivo experiments

All experiments were conducted in compliance with the UK Home Office-approved project licences and personal licences (Animals Scientific Procedures Act 1986) and within institutional welfare guidelines (Francis Crick Institute). Animal experiments were subject to ethical review by the Francis Crick Animal Welfare and Ethical Review Body and carried out under UK Home Office project license P319AE968. Mice (3–5 per cage) had ad libitum access to food and water and were kept in a 12 h day/night cycle (7:00 to 19:00) in rooms at 21 °C at 55% humidity. Mice were acclimatised to their environment for at least one week prior to experimentation. Mice used in the experiments were littermates randomly assigned to experimental groups. The experimental diets used in Fig. 4F (control diet and –SG diet) were previously described as “Diet 1-control” and “Diet 1-SG-free”^7^. Control diet contains all essential amino acids in addition to serine, glycine, glutamine, arginine, cysteine and tyrosine. The SG- diet is formulated as the control diet lacking serine and glycine which is compensated by a proportionally increased level of the other amino acids to reach the same total amino acid content.

### Xenograft experiments

In Fig. 4D, E and Supplementary Fig. 4D, CD-1 nude mice (obtained from Charles River, 7-9 weeks old) received a subcutaneous injection of 4 × 10^6^ DLD-1 cells suspended in PBS. Mice were placed on experimental diet two days after tumour injections. A further two days after diet change, mice were treated either with vehicle (0.5% methylcellulose (Sigma, H7509), 0.5% Tween-80 (Sigma, P8192) or PH755 (obtained by Raze Therapeutics) prepared in vehicle once daily by oral gavage. The starting dosage of PH755 was 100 mg/kg (for seven days) and was subsequently lowered to 50 mg/kg (for six days) and increased again to 75 mg/kg (for seven days). This experiment was generated for a previously published experiment^9^.

In Supplementary Fig. 4C, NOD.C-Prkdc^SCID^ Il2rg^tm1Wjl^/SzJ (NSG) mice in between 8 and 12 weeks old (obtained from The Francis Crick Institute breeding facility) received subcutaneous injection of 4 × 10^6^ DLD-1 cells suspended in PBS and left to grow for 4 weeks before harvesting the tumours.

### Statistical analysis

All statistical analyses were performed using GraphPad Prism 8 software. Unpaired Student’s *t* test was performed to compare two groups to each other. If the variance, determined by the F test, between the two groups was unequal, a Welch’s correction was applied. For multiple comparison, a one-way ANOVA was used. If the variance between groups, determined by the Brown–Forsythe test, was unequal, a Brown–Forsythe and Welch correction was applied. *p* value below 0.05 was considered statistically significant. Significance is indicated as follows: *p < 0.05, **p < 0.01, ***p < 0.001, ****p < 0.0001, ns no significance. All measurements were taken from distinct samples, as noted in figure legends, and no data were excluded. Sample sizes were based in standard protocols in the field, metabolic data were assigned in a random order before analysis by LC–MS, mice were randomly assigned to a treatment and mouse experiments were blinded to the person performing metabolomic analysis.

### Reporting summary

Further information on research design is available in the Nature Research Reporting Summary linked to this article.

## Supplementary information

Supplementary Information

Reporting Summary

## Data Availability

All the data (including source data) supporting the findings of this study are available within the article, the supplementary information files and the source data file. Source data are provided with this paper.

## Source data

Source Data
