# Supplementary material for: The impact of physiological metabolite levels on serine uptake, synthesis and utilization in cancer cells
Source: Nat Commun. 2021 Oct 26;12:6176. doi: 10.1038/s41467-021-26395-5 (PMC8548528; doi:10.1038/s41467-021-26395-5)
Supplement: Supplementary file 1 — Supplementary Information [file 41467_2021_26395_MOESM1_ESM.pdf]

Supplementary Information

The impact of physiological metabolite levels on serine uptake, synthesis and utilization in cancer cells.

Supplementary Table 1

Gene	IDT Reference	Primer 1	Primer 2	Probe
ASCT1	Hs.PT.58.4102571	CAT CCC CTT CCA CAT TCA CC	CCT CAC CAT TGC CAT TAT CCT	/56-FAM/TCC ACA ATC /ZEN/CAG TCC ACA GCC AG/3IABkFQ/
ASCT2	Hs.PT.58.21358468	CCA TTA TTC TCC TCC ACG CA	CCT CAT CTA CTT CCT CTT CAC C	/56-FAM/CCA CGC TGC /ZEN/CGC TGA TGA TG/3IABkFQ/
POLR2A	Hs.PT.39a.19639531	TCG TCT CTG GGT ATT TGA TGC	CAG TTC GGA GTC CTG AGT C	/56-FAM/ACT GAA GCG /ZEN/AAT GTC TGT GAC GGA G/3IABkFQ/

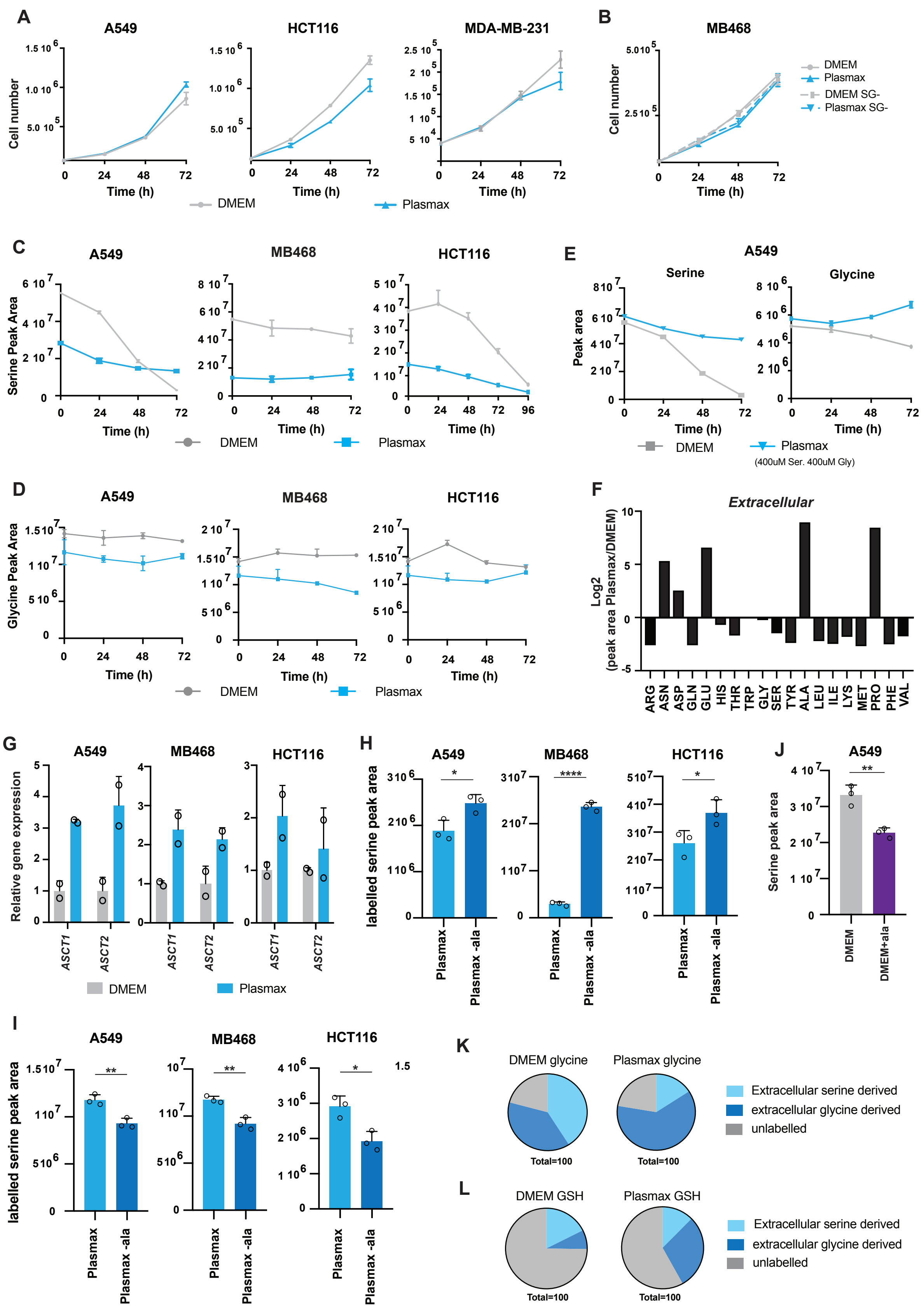

A
A549
HCT116
MDA-MB-231
B
MB468
Cell number
Time (h)
DMEM
Plasmax
DMEM SG-
Plasmax SG-
C
Serine Peak Area
E
A549
Serine
Glycine
Peak area
(400uM Ser. 400uM Gly)
D
Glycine Peak Area
F
Extracellular
Log2 (peak area Plasmax/DMEM)
ARG ASN ASP GLN GLU HIS THR TRP GLY SER TYR ALA LEU ILE LYS MET PRO PHE VAL
G
Relative gene expression
ASCT1
ASCT2
H
labelled serine peak area
Plasmax
Plasmax -ala
J
Serine peak area
DMEM+ala
I
K
DMEM glycine
Plasmax glycine
Total=100
Extracellular serine derived
extracellular glycine derived
unlabelled
L
DMEM GSH
Plasmax GSH

Supplementary Figure 1: (**A**) Growth curve of cells grown in complete DMEM (grey) or Plasmax (blue). (**B**) Growth curve of serine deprivation-resistant MDA-MB-468 in DMEM (grey) or Plasmax (blue) lacking or not serine and glycine (SG-). (**C**) Extracellular levels of serine measured by LC-MS in supernatants of cells grown in DMEM (grey) or Plasmax (blue). (**D**) Extracellular levels of glycine measured by LC-MS in supernatants of cells grown in DMEM (grey) or Plasmax (blue). (**E**) Extracellular levels of serine and glycine measured by LC-MS of cells grown in DMEM (grey) or Plasmax (400µM SER, 400µM GLY) (blue) modified to contain the same levels of serine and glycine present in DMEM (400 µM of serine and glycine). (**F**) Representation of the ratio of the amino acid peak areas normalized to tryptophan (present at the same concentration in both media) in Plasmax/DMEM measured by LC-MS. (**G**) RT-qPCR of *ASCT1 (SLC1A4*) and *ASCT2 (SLC1A5)* in cells grown in DMEM (grey) or Plasmax (blue) for 72 h. (**H**) Intracellular levels of 2,3,3-D_3-serine measured by LC-MS after cells were grown 24 h in Plasmax (light blue) or Plasmax depleted in alanine (dark blue) (unpaired Student *t* test, *p<0.05, ****p<0.0001). (**I**) Extracellular levels of $^{13}C_3$ ^{15}N-Serine measured by LC-MS after cells were grown 24h in Plasmax (light blue) or Plasmax depleted in Alanine (dark blue) (unpaired Student *t* test, *p<0.05, **p<0.01). (**J**) Intracellular levels of serine measured by LC-MS after cells were grown 24 h in DMEM (grey) or DMEM supplemented with 500 µM alanine (purple) (unpaired Student *t* test, **p<0.01). (**K**) A549 cells were separately incubated with 400 µM or 140 µM of 2,3,3-D_3-serine and 400 µM or 330 µM of 2,2-D_2-glycine for 4h in DMEM or Plasmax, respectively. Percentage of glycine derived from 2,3,3,-D_3-serine and extracellular 2,2-D_2-glycine were plotted as parts of whole. (**L**) A549 cells were separately incubated with 400 µM or 140 µM of 2,3,3-D_3-serine and 400 µM or 330 µM of 2,2-D_2-glycine for 4h in DMEM or Plasmax, respectively. Percentage of glutathione derived from extracellular 2,3,3-D_3-serine and extracellular 2,2-D_2-glycine were plotted as parts of whole. Data are represented as mean ± SD of triplicate wells and are representative of 3 independent experiments. Supplementary Figure 1G is represented as mean ± SD of duplicate wells and is representative of 3 independent experiments.

Source data are provided as a Source Data file.

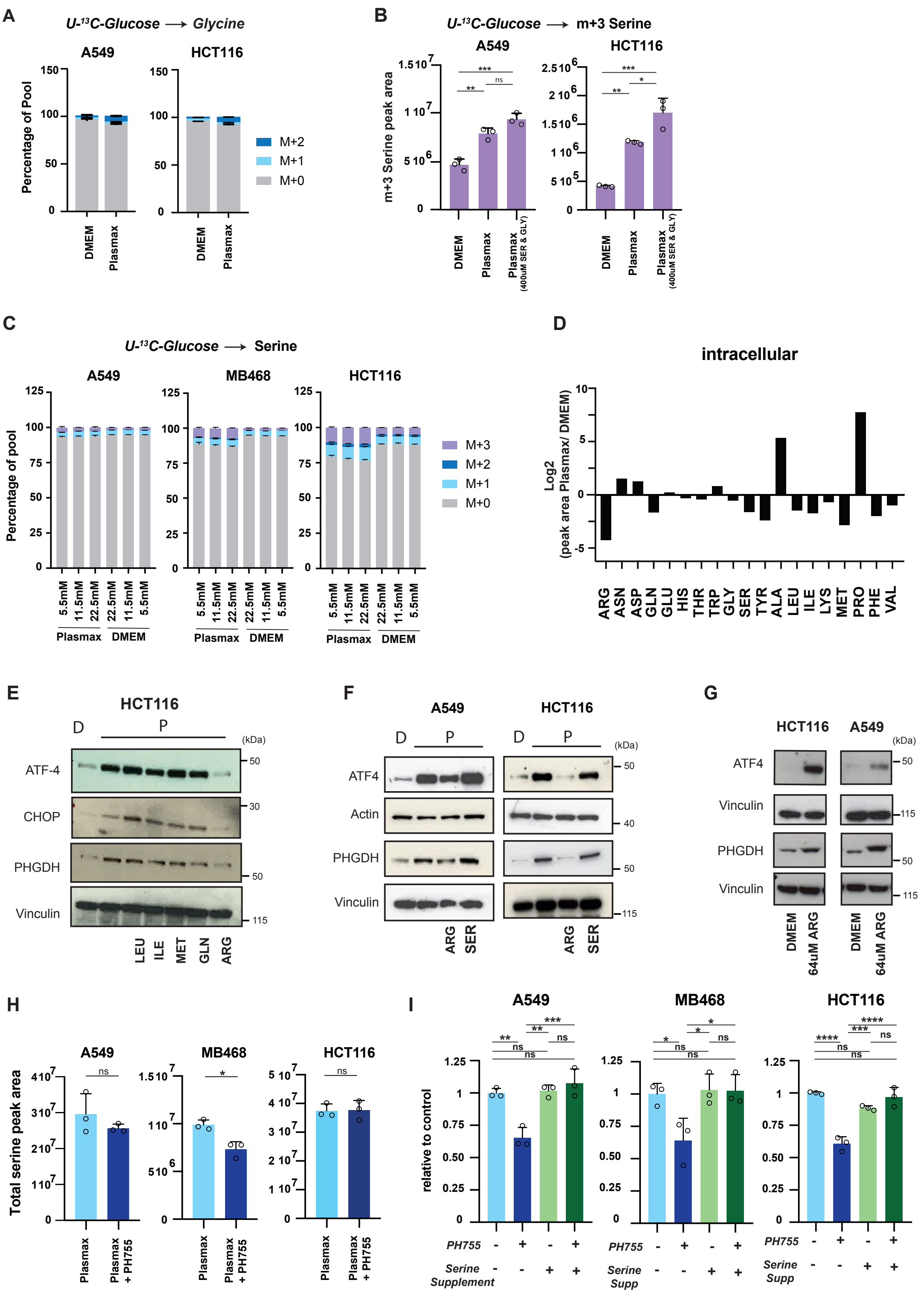
A
U-13C-Glucose → Glycine
A549
HCT116
Percentage of Pool
M+2
M+1
M+0
DMEM
Plasmax
B
U-13C-Glucose → m+3 Serine
m+3 Serine peak area
DMEM
Plasmax
Plasmax (400uM SER & GLY)
C
U-13C-Glucose → Serine
MB468
Percentage of pool
M+3
5.5mM
11.5mM
22.5mM
D
intracellular
Log2 (peak area Plasmax/ DMEM)
ARG ASN ASP GLN GLU HIS THR TRP GLY SER TYR ALA LEU ILE LYS MET PRO PHE VAL
E
ATF-4
CHOP
PHGDH
Vinculin
(kDa)
F
ATF4
Actin
G
64uM ARG
H
Total serine peak area
Plasmax + PH755
I
relative to control
PH755
Serine Supplement
Serine Supp

Supplementary Figure 2: (**A**) Percentage of pool of intracellular glycine measured by LC-MS after incubation for 4 h with 22.5 mM or 5.5 mM of U-^{13}C-glucose in DMEM or Plasmax, respectively. Data are represented as mean ± SD of triplicate wells. (**B**) Intracellular levels of M+3 serine of cells grown in DMEM, Plasmax or Plasmax (400µM SER, 400µM GLY) and incubated 4 h with 22.5 mM, 5.5 mM or 5.5 mM of U-^{13}C-glucose in DMEM, Plasmax or Plasmax (400µM SER, 400µM GLY) respectively. Data are represented as mean ± SD of triplicate wells (Multiple comparison by one-way Anova, *p<0.05, **p<0.01, ***p<0.001, ns: no significance. (**C**) Percentage of pool of intracellular M+3 serine measured by LC-MS after incubation for 4 h with different concentrations of U-^{13}C-glucose in DMEM or Plasmax. Data are represented as mean ± SD of triplicate wells. (**D**) Representation of the ratio of intracellular amino acid levels in Plasmax/DMEM measured by LC-MS. (**E**) Western blot of HCT116 cells grown for 24h in DMEM (D) or Plasmax (P) supplemented with 630 µM leucine (LEU), 660µM isoleucine (ILE), 170µM methionine (MET), 1350µM glutamine (GLN) and 436µM arginine (ARG). Membrane was cut and ATF4, PHGDH and CHOP were probed and membrane was reprobed with vinculin as loading control. (**F**) Western blot of cells grown for 24 h in DMEM (D) or Plasmax (P) supplemented with 436 µM of arginine (ARG) or 260 µM of serine (SER) in order to recapitulate DMEM levels of arginine (500 µM) or serine (400 µM). Membranes were reprobed with Actin for ATF-4 blot and Vinculin for PHGDH blot as loading control. (**G**) Western blot of cells grown for 24 h in DMEM or DMEM 64 µM of arginine (64 µM ARG). Membrane was reprobed with vinculin as loading control. (**H**) Total serine peak area was measured in cells that were incubated 24h in Plasmax (light blue) alone or treated with 10µM of PH755 (dark blue). Data are represented as mean ± SD of triplicate wells (unpaired Student *t* test, *p<0.05, ns: no significance). (**I**) Growth fold change relative to the full Plasmax condition (PH755-/Serine Supp -) after 72 h of proliferation in the different conditions. When indicated cells were treated with 10µM of PH755 and supplemented with 400µM of exogenous serine. PH755-/Serine Supp- (light blue), PH755+/Serine Supp- (dark blue), PH755-/Serine Supp+ (light green), PH755+/Serine Supp+ (dark green). Data are represented as mean ± SD of triplicate wells (Multiple comparison by one-way Anova, *p<0.05, **p<0.01, ***p<0.001, ****p<0.0001). All graph are representative of 3 independent experiments and blots are representative of 2 independent experiments.

Source data are provided as a Source Data file.

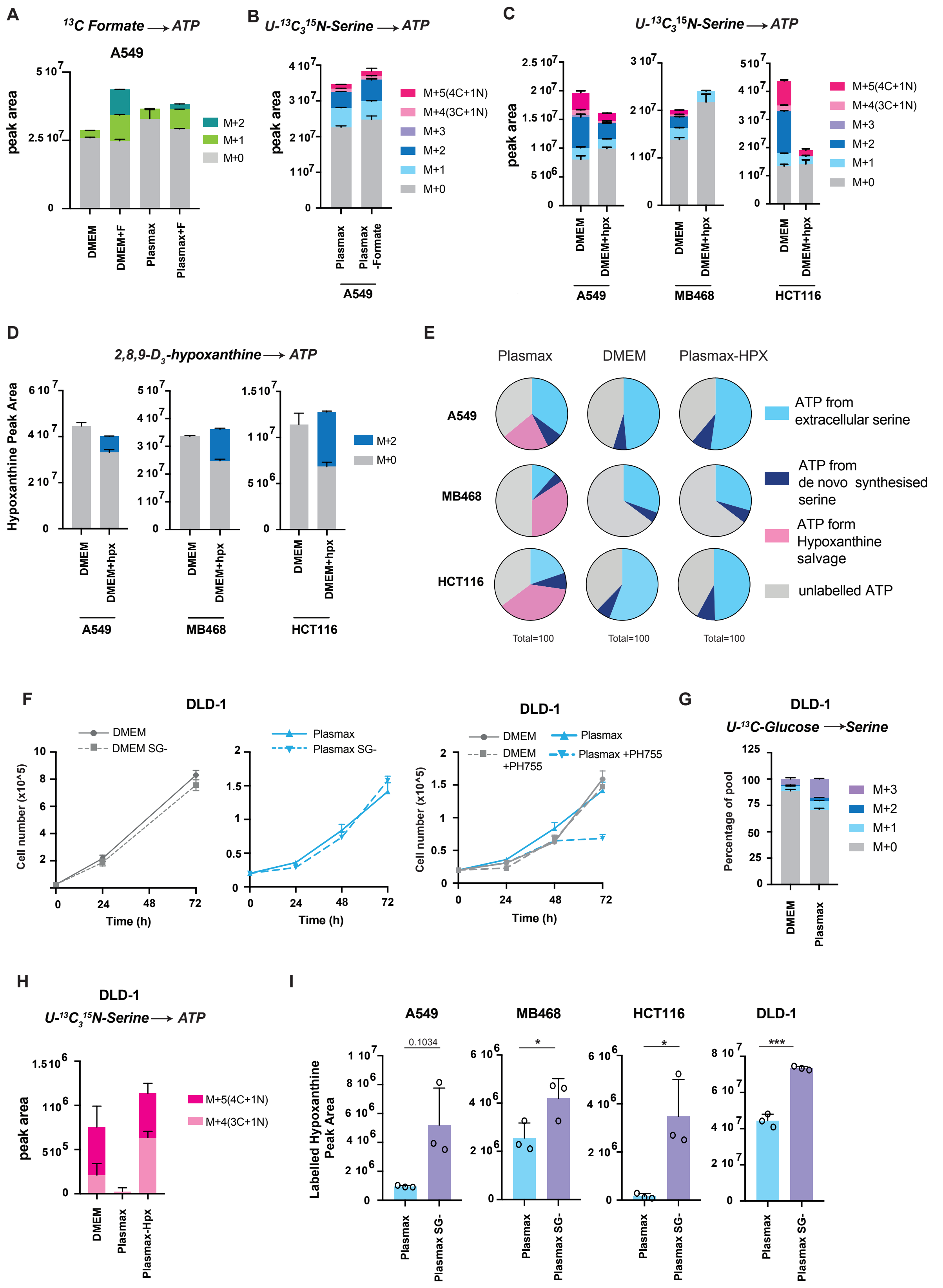

A
^{13}C Formate → ATP
A549
peak area
M+2
M+1
M+0
DMEM
DMEM+F
Plasmax
Plasmax+F
B
U-^{13}C$_3$^{15}N-Serine → ATP
M+5(4C+1N)
M+4(3C+1N)
M+3
M+2
M+1
M+0
Plasmax
Plasmax -Formate
C
DMEM
DMEM+hpx
A549
MB468
HCT116
D
2,8,9-D$_3$-hypoxanthine → ATP
Hypoxanthine Peak Area
E
Plasmax
DMEM
Plasmax-HPX
ATP from extracellular serine
ATP from de novo synthesised serine
ATP form Hypoxanthine salvage
unlabelled ATP
Total=100
F
DLD-1
DMEM SG-
Plasmax SG-
DMEM +PH755
Plasmax +PH755
Cell number (x10^5)
Time (h)
G
U-^{13}C-Glucose → Serine
Percentage of pool
H
Plasmax-Hpx
I
Labelled Hypoxanthine Peak Area
0.1034
*
***

Supplementary Figure 3: (**A**) Intracellular levels of ATP measured by LC-MS in cells incubated for 4 h with 33 µM of ^{13}C-formate (+F). In cells grown in Plasmax, non-labelled formate was removed and replaced by ^{13}C-formate (+F). Data are represented as mean ± SD of triplicate wells. (**B**) Intracellular levels of ATP measured by LC-MS in cells grown in Plasmax and deprived or not of formate and incubated for the last 4 h with 140 µM of $^{13}C_3$-^{15}N-serine. Data are represented as mean ± SD of triplicate wells. (**C**) Intracellular levels of ATP after cells were grown in DMEM and DMEM supplemented with 5 µM hypoxanthine and incubated for 4 h with 400 µM of $^{13}C_3$-^{15}N-serine. Data are represented as mean ± SD of triplicate wells. (**D**) Intracellular levels of ATP in cells incubated for 4 h with or without 5 µM 2,8,9-D_3-hypoxanthine in DMEM. Data are represented as mean ± SD of triplicate wells (**E**) Cells were incubated separately for 4 h with 22.5 mM or 5.5 mM of U-^{13}C-glucose, 400 µM or 140 µM of $^{13}C_3$-^{15}N-serine and 0 µM or 5 µM of 2,8,9-D3-hypoxanthine in DMEM and Plasmax, respectively. Percentage of pools of ATP derived from U-^{13}C-glucose-derived serine (ATP+4/+6/+7/+8), extracellular $^{13}C_3$-^{15}N-serine (ATP+1/+2/+3/+4/+5) and 2,8,9-D3-hypoxanthine were plotted as parts of whole. (**F**) Growth curve of DLD-1 cells grown in DMEM (grey), DMEM SG-, Plasmax (blue) and Plasmax SG- or DMEM and Plasmax with or without 10 µM of PH755. Data are represented as mean ± SD of triplicate wells. (**G**) Percentage of pool of intracellular serine measured by LC-MS after incubation for 4h with 22.5 mM or 5.5 mM of U-^{13}C-glucose in DMEM or Plasmax, respectively. Data are represented as mean ± SD of triplicate wells. (**H**) Intracellular levels of ATP measured by LC-MS after incubation for 4 h with 400 µM, 140 µM or 140 µM of $^{13}C_3$-^{15}N-Serine in DMEM, Plasmax or Plasmax-hpx, respectively. Data are represented as mean ± SD of triplicate wells. (**I**) Intracellular levels of 2,8,9-D_3-hypoxanthine measured by LC-MS. Cells were grown 24 h in the different media conditions, Plasmax (blue) and Plasmax SG- (purple), before incubation with 5 µM of 2,8,9-D_3-hypoxanthine for the last 4 h. Data are represented as mean ± SD of triplicate wells (unpaired Student *t* test for DLD-1 and MB468, Welch correction was applied for A549 and HCT116 *$p<0.05$, ***$p<0.001$). All panels are representative of 3 independent experiments. Source data are provided as a Source Data file.

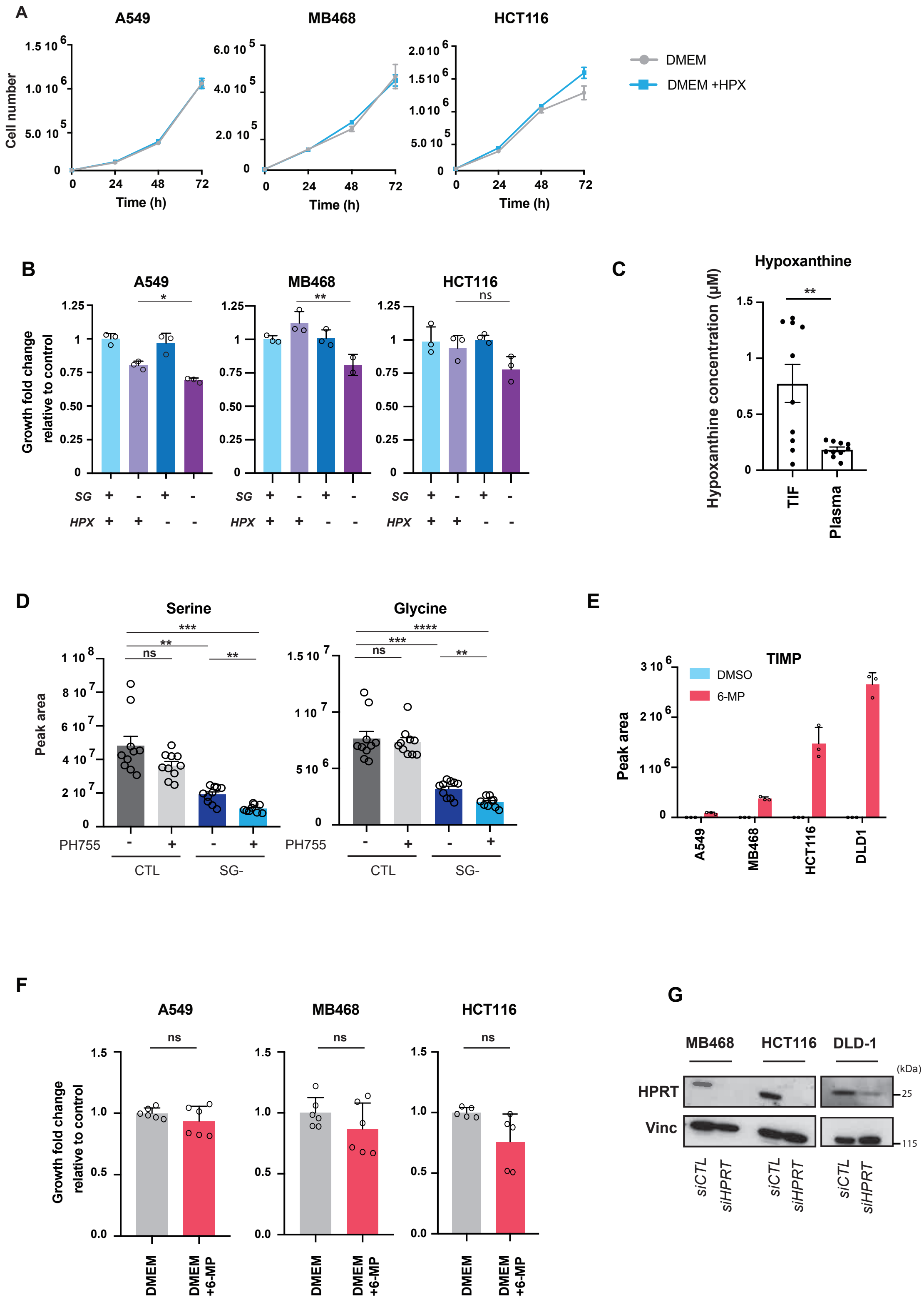
A
A549
MB468
HCT116
Cell number
Time (h)
DMEM
DMEM +HPX
B
Growth fold change relative to control
SG
HPX
C
Hypoxanthine
Hypoxanthine concentration (μM)
TIF
Plasma
D
Serine
Glycine
Peak area
PH755
CTL
SG-
E
TIMP
DMSO
6-MP
DLD1
F
DMEM
DMEM +6-MP
G
MB468
HCT116
DLD-1
HPRT
Vinc
(kDa)
siCTL
siHPRT

Supplementary Figure 4: (**A**) Growth curve of cells grown in complete DMEM (grey) or DMEM supplemented with 5 µM hypoxanthine (+HPX) (blue). Data are represented as mean ± SD of triplicate wells and representative of 3 independent experiments. (**B)** Growth fold change relative to the full Plasmax condition (SG+/HPX+) after 72 h of proliferation in the different conditions. SG+/HPX+ (light blue), SG-/HPX+ (light purple), SG+/HPX- (dark blue), SG-/HPX- (dark purple). Data are represented as mean ± SD of triplicate wells and representative of 3 independent experiments (Direct comparison by unpaired Student *t* test, *p<0.05, **p<0.01, ns: no significance). (**C**) Hypoxanthine quantification in plasma and tumour interstitial fluid (TIF) from NSG mice with DLD-1 cell xenograft measured by LC-MS. Data are represented as mean ± SEM of 10 mice (unpaired Student *t* test with Welch correction *p<0.05). (**D**) Levels of serine and glycine measured by LC-MS in plasma collected at end-point from DLD-1 xenograft bearing mice fed with control with vehicle (n=10) (light grey) or PH755 (n=10) (dark grey) or serine/glycine free diet with vehicle (n=10) (dark blue) or PH755 (n=9) (light blue). Data are represented as mean ± SEM (Multiple comparison by one-way Anova with Brown-Forsythe and Welch correction, *p<0.05, **p<0.01, ***p<0.001, ****p<0.0001, ns: no significance). These data have been previously published in Tajan et al, 2021 (doi: 10.1038/s41467-020-20223-y). (**E**) 6-thio-Iosinic acid (TIMP) levels after 2 hour incubation with 2.5µM of 6-MP (red) or DMSO (blue). Data are represented as mean ± SD of triplicate wells and representative of two independent experiments. (**F**). Growth fold change relative to DMEM control condition (grey) after 72 h of proliferation. Cells were treated with 2.5uM of 6-MP (red). Data are represented as mean ± SD and pooled form two independent experiments containing 2 to 3 replicate wells (unpaired Student *t* test for MB468 and with Welch correction for A549 and HCT116, ns: no significance). (**G**) Cells were transfected with siHPRT1 or non-targeting siRNA. Protein lysate was harvested at 72h after transfection. Membrane was reprobed with vinculin as loading control. Blots are representative of 3 independent experiments.

Source data are provided as a Source Data file.
