# Supplementary material for: The impact of physiological metabolite levels on serine uptake, synthesis and utilization in cancer cells
Source: Nat Commun. 2021 Oct 26;12:6176. doi: 10.1038/s41467-021-26395-5 (PMC8548528; doi:10.1038/s41467-021-26395-5)
Supplement: Supplementary file 2 — Reporting Summary [file 41467_2021_26395_MOESM2_ESM.pdf]

nature research

Corresponding author(s): Karen Vousden

Last updated by author(s): Sep 8, 2021

Reporting Summary

Nature Research wishes to improve the reproducibility of the work that we publish. This form provides structure for consistency and transparency in reporting. For further information on Nature Research policies, see our Editorial Policies and the Editorial Policy Checklist.

Statistics

For all statistical analyses, confirm that the following items are present in the figure legend, table legend, main text, or Methods section.

n/a	Confirmed	
☐	☒	The exact sample size (*n*) for each experimental group/condition, given as a discrete number and unit of measurement
☐	☒	A statement on whether measurements were taken from distinct samples or whether the same sample was measured repeatedly
☐	☒	The statistical test(s) used AND whether they are one- or two-sided<br>*Only common tests should be described solely by name; describe more complex techniques in the Methods section.*
☒	☐	A description of all covariates tested
☒	☐	A description of any assumptions or corrections, such as tests of normality and adjustment for multiple comparisons
☐	☒	A full description of the statistical parameters including central tendency (e.g. means) or other basic estimates (e.g. regression coefficient) AND variation (e.g. standard deviation) or associated estimates of uncertainty (e.g. confidence intervals)
☐	☒	For null hypothesis testing, the test statistic (e.g. *F*, *t*, *r*) with confidence intervals, effect sizes, degrees of freedom and *P* value noted<br>*Give P values as exact values whenever suitable.*
☒	☐	For Bayesian analysis, information on the choice of priors and Markov chain Monte Carlo settings
☒	☐	For hierarchical and complex designs, identification of the appropriate level for tests and full reporting of outcomes
☒	☐	Estimates of effect sizes (e.g. Cohen's *d*, Pearson's *r*), indicating how they were calculated

Our web collection on statistics for biologists contains articles on many of the points above.

Software and code

Policy information about availability of computer code

Data collection	For NMR: For 1H 1D profiling spectra the Bruker pulse program zgesgppe (avance-version 13/08/01) for excitation sculpting with pure echo (Adams R.W, et al. Chem Commun (Camb), 2013. 49(4): p.358-360) was used with 20 ppm sweep width, 1 s relaxation delay and 4 s acquisition time.<br>RT-qPCR data were collected on a QuantStudio 3 system from applied biosystems.
Data analysis	For NMR: Data were processed and analysed using Chenomx NMR Suite (version 8.6) (Chenomx, Edmonton, Canada).<br>For LC-MS: Data were analysed with TraceFinder (version 4.1) and Xcalibur (verison 4.0.27.19) from Thermofisher.<br>Excel (16.43); GraphPad prism 8 (version 8.0.0)

For manuscripts utilizing custom algorithms or software that are central to the research but not yet described in published literature, software must be made available to editors and reviewers. We strongly encourage code deposition in a community repository (e.g. GitHub). See the Nature Research guidelines for submitting code & software for further information.

Data

Policy information about availability of data

All manuscripts must include a data availability statement. This statement should provide the following information, where applicable:

- Accession codes, unique identifiers, or web links for publicly available datasets
- A list of figures that have associated raw data
- A description of any restrictions on data availability

The source data from the principal and supplementary figures are provided as a source data file or are available in the supplementary information file. Other data that support the findings in this study are available from the corresponding author upon reasonable request.

Field-specific reporting

Please select the one below that is the best fit for your research. If you are not sure, read the appropriate sections before making your selection.

- [x] Life sciences
- [ ] Behavioural & social sciences
- [ ] Ecological, evolutionary & environmental sciences

For a reference copy of the document with all sections, see nature.com/documents/nr-reporting-summary-flat.pdf

Life sciences study design

All studies must disclose on these points even when the disclosure is negative.

Sample size	Experiments were performed using sample sizes based on standard protocols in the field (see Diehl et al. Nat Metab. 2019 Sep;1(9):861-867, Luengo et al. Nat Commun. 2019 Dec 6;10(1):5604). No statistical test was performed to predetermine sample size. All mouse experiments started with 10 mice per group but occasionally a mouse had to be euthanized before the end of the experiment to comply with the animal welfare regulations.
Data exclusions	No data were excluded
Replication	Information provided in Figure legends
Randomization	All metabolic data are assigned a random order before being injected through the LC-MS column.<br>For mouse experiments with treatment, cages of 5 mice grafted with tumours were randomly assigned a treatment.<br>For other experiments (Western blot, RT-qPCR and proliferation assays) no randomization was possible.
Blinding	Mouse experiments were blinded for both the collection and analysis of the metabolomics data.

Reporting for specific materials, systems and methods

We require information from authors about some types of materials, experimental systems and methods used in many studies. Here, indicate whether each material, system or method listed is relevant to your study. If you are not sure if a list item applies to your research, read the appropriate section before selecting a response.

Materials & experimental systems

n/a	Involved in the study	
[ ]	[x]	Antibodies
[ ]	[x]	Eukaryotic cell lines
[x]	[ ]	Palaeontology and archaeology
[ ]	[x]	Animals and other organisms
[x]	[ ]	Human research participants
[x]	[ ]	Clinical data
[x]	[ ]	Dual use research of concern

Methods

n/a	Involved in the study	
[x]	[ ]	ChIP-seq
[x]	[ ]	Flow cytometry
[x]	[ ]	MRI-based neuroimaging

Antibodies

Antibodies used	PHGDH: Cell signalling, #13428, polyclonal, lot 1<br>ATF4: Cell signalling, #11815, clone D4B8, lot 5<br>CHOP: Cell signalling, #2895, clone L63F7, lot 11<br>PSAT: Abcam, ab96136, polyclonal, lot GR265773-22<br>PSPH: Abcam, ab96414, polyclonal, lot GR8276-8<br>Actin: Abcam, ab8226, mAbcam 8226 , lot GR324494-3<br>Vinculin: Santa-Cruz, sc-73614, clone 7F9, lot B0717<br>HPRT: Abcam, Ab133242, EPR5298, lot GR97370-7
Validation	All antibodies described in this study were verified and confirmed for species reactivity and application as per the manufacturers' disclosure.<br><br>Western blot:<br><br>PHGDH (13428) from Cell Signaling Technology. Species reactivity: multiple species including human, validated for Western blot by manufacturer: https://www.cellsignal.com/products/primary-antibodies/phgdh-antibody/13428?Ntk=Products&Ntt=13428

ATF-4 (D4B8) (11815) from Cell Signaling Technology. Species reactivity: multiple species including human, validated for Western blot by manufacturer: https://www.cellsignal.com/products/primary-antibodies/atf-4-d4b8-rabbit-mab/11815?Ntk=Products&Ntt=11815

CHOP (L63F7) (2895) from Cell Signaling Technology. Species reactivity: multiple species including human, validated for Western blot by manufacturer: https://www.cellsignal.co.uk/products/primary-antibodies/chop-l63f7-mouse-mab/2895

PSAT (ab96136) from Abcam. Species reactivity: human, validated for Western blot by manufacturer: https://www.abcam.com/phosphoserine-aminotransferase-antibody-ab96136.html

PSPH (ab96414) from Abcam. Species reactivity: human, validated for Western blot by manufacturer: https://www.abcam.com/psphantibody-ab96414.html

Vinculin (7F9) (sc-73614) from Santa Cruz Biotechnology. Species reactivity: multiple species including human, validated for Western blot by manufacturer: https://datasheets.scbt.com/sc-73614.pdf

HPRT (EPR5298) Ab133242 from Abcam. Species reactivity: multiple species including human, validated for Western blot by manufacturer: https://www.abcam.com/hprt-antibody-epr5298-ab133242.html

Actin (mAbcam 8226) Ab8226 from Abcam.pecies reactivity: multiple species including human, validated for Western blot by manufacturer: https://www.abcam.com/beta-actin-antibody-mabcam-8226-loading-control-ab8226.html

Eukaryotic cell lines

Policy information about cell lines

Cell line source(s)	All cell lines (A549, MDA-MB-468, MDA-MB-231, HCT116 and DLD-1) were provided by the Cell Services depository for the Francis Crick Institute
Authentication	All cell lines were authenticared using STR profiling and species identificationes.
Mycoplasma contamination	All cell lines were negative for mycoplasma upon thawing.
Commonly misidentified lines (See ICLAC register)	No commonly misidentified cell lines were used in the study.

Animals and other organisms

Policy information about studies involving animals; ARRIVE guidelines recommended for reporting animal research

Laboratory animals	Fig4D, Fig4E and FigS4D, female CD-1 nude mice aged in between 7 and 9 weeks were obtained at Charles River.<br>Fig S4C Mix of female and male NSG mice in between 8 and 12 weeks obtained by the Francis Crick Institute breeding facility.
Wild animals	This study did not involve wild animals.
Field-collected samples	This study did not involve samples collected from the field.
Ethics oversight	All experiments were conducted in compliance with the UK Home Office-approved project licences and personal licences (Animals Scientific Procedures Act 1986) and within institutional welfare guidelines (Francis Crick Institute).

Note that full information on the approval of the study protocol must also be provided in the manuscript.
